# Starvation and cadmium affect energy reserves and oxidative stress in individuals of *Spodoptera exigua*

**DOI:** 10.1007/s10646-022-02588-6

**Published:** 2022-09-29

**Authors:** Anna Pompka, Elżbieta Szulińska, Alina Kafel

**Affiliations:** Department of Natural Sciences, University of Silesia in Katowice, Institute of Biology, Biotechnology and Environmental Protection, Bankowa 9, PL 40-007 Katowice, Poland

**Keywords:** Metal, Starvation, Herbivore insect, Energy biomolecules, Free radicals

## Abstract

Different factors, such as starvation and metal exposure, may affect development and cause oxidative stress in insects. Some host plants may contain a high concentration of cadmium due to their hyperaccumulating property. The negative effects of metals and hunger may be manifested by low availability of energetic substrates. This study aimed to assess whether the insect population with a history of long metal exposure may better manage metal stress or/and starvation at different developmental stages, with the use of energetic substrates. Two strains of *Spodoptera exigua* model organism were tested: control strain and cadmium strain (treated continuously for over 200 generations with subtoxic amounts of cadmium). The effects of different factors, individually and in combination, on the tested strains were assessed, first by determining the body weight of larvae and pupae and then by estimating the concentration of biomolecules (proteins, carbohydrates, lipids, or glycogen) in the 4th and 5th larval stages and in pupae, and the total antioxidant capacity and lipid peroxidation level in the 4th larval stage. Compared to control strain, cadmium strain individuals exhibited changes in the concentration of soluble carbohydrates and protein. This was partly related to earlier 1-day starvation. In particular, changes in carbohydrate concentration seemed to be a sensitive biomarker of metal stress, independent of the age of individuals and period of starvation. However, the increase in the total antioxidant capacity and the concentration of lipid peroxidation products in the 4th larval stage under the effect of cadmium was dependent on strain origin.

## Highlights

- Role of carbohydrates as a sensitive metal and hunger stress biomarker.
- Higher oxidative stress in metal-exposed insects regardless of starvation treatment.
- Slightly marked effect of 1-day starvation on lipid concentration in larvae and pupae.

## Introduction

Insects are exposed to a variety of stressors. Among them, heavy metals and short period of starvation (lack of food) may have a detrimental effect on the development of insects and increase oxidative stress in their tissues. Recent studies highlighted some starvation consequences on different developmental stages of herbivorous pests; e.g., larvae or imago (Wheeler et al. 2001; Chen and Ruberson 2008). The findings observed after starvation period indicated its negative consequences for further development of insects. Hunger initially causes an increase in the search for food activity, but after a while the activity of insects decreases, due to a reduced metabolic rate. This could be accompanied by an increase in aggression, rivalry, and cannibalism (Scharf 2016). It has been shown that hunger can prolong the development of larvae and lead to a significant reduction in pupae weight, as well as in the fertility of females (Chen and Ruberson 2008; Singh et al., 2021). The other effects of starvation may include greater susceptibility to parasites and pathogens (Yang et al. 2007). In a study investigating the effects of hunger and exposure to plant extracts in *Spodoptera litura* (Lepidoptera, Noctuidae) larvae, Wheeler et al. (2001) found one or two additional larval stages of this specimen. As with other representatives of the Noctuidae family, starvation of larvae in laboratory conditions was associated with a prolonged larval period and a higher mortality rate (Tignor and Eaton 1986). It was also noted that, depending on the age of the examined individuals at which starvation (lasted 48 h) occurred, the mass gained by the tested individuals differed. At various larval stages of *S. exigua* (1st, 3rd, and 5th), and after the starvation period during earlier larval development, the pupae collected were heavier, probably because of the energy expenditure during the extended period of development. When hunger occurred at the last larval stage, the pupae had a reduced body weight, which was likely due to their inability to compensate for the loss suffered during hunger (Chen and Ruberson 2008). A study showed that two different species of Lepidoptera responded differently to the lack of food. The tested *Grammia geneura* (Arctiidae) larvae were characterized by a lower mortality rate than *Manduca sexta* (Sphingidae) larvae. This was probably due to the low rate of water loss and the ability to decrease the rate of metabolism (Woods and Singer 2001). In another study, changes in the levels of energetic substrates, such as trehalose and glycogen, in relation to the duration of hunger were determined in starved adult ladybugs *Harmonia axyridis* (Coleoptera, Coccinellidae). During the first 8 h of starvation, the level of trehalose was found to be decreased, while glycogen remained at the same level. From 24 to 72 h of starvation, a significant decrease in trehalose level was noted. At the end of starvation, an increase in trehalose activity and relative gene expression was observed (Shi et al. 2017). It has been demonstrated that reduction of glucose in hemolymph in starving *S. litura* individuals caused a likely increase in glucose concentration and glycogen phosphorylase activity in the fat body (Tzeng 2001). A higher intensity of apoptosis was observed with a deficiency of certain substances in food in *S. litura*. For example, glucose deficiency led to the formation of apoptotic bodies in the cells of these insects. After 48 h, this effect started to diminish, although the apoptosis process was still ongoing (Liu et al. 2007).

One of the indicators of metal toxicity may be oxidative stress and enhanced cost of antioxidant defense. A study determined the activity of the detoxifying enzyme glutathione S-transferase (GST) in the larvae from the first and second generations of *S. exigua* insects treated with zinc- and cadmium-supplemented medium. The authors noted an increase in GST activity in the fat body and Malpighi tubules caused by zinc and a reduction in enzyme activity under influence of cadmium (Kafel et al. 2003). The exposure of last instar *S. exigua* larvae to cadmium in the diet resulted in lower larval survival rate and prolonged larval stages. Cadmium also contributed to an increase in total antioxidant capacity (TAC) and oxidized glutathione level in the larval hemolymph (Kafel et al. 2012).

Research on long-term, multigenerational exposure of *S. exigua* populations to cadmium provided some evidence for better tolerance of these insects to this metal. It was manifested, among others, by better survival rate in the presence of cadmium compared to populations originating from the control strain (Kafel et al. 2012, 2014; Płachetka-Bożek et al. 2018). It was also observed that individuals from cadmium and control strains showed differences in the induction of HSP70 proteins, which play an important role in protecting cells from chronic stress. However, larvae from metal-selected strains were characterized by less susceptibility to pesticides (Augustyniak et al. 2017; Tarnawska et al. 2019). The latter also showed more efficient DNA repair mechanisms in cells (Augustyniak et al. 2016). Maintenance of the oxidation–antioxidant balance may depend on the developmental stage, as shown in the case of *Lymantria dispar* larvae (from the 3rd to the 6th stage), as well as contribute to increased metabolic rate. The presence of cadmium in the medium also prolonged the duration of larval stages and decreased the body weight of the larvae (Mirčić et al. 2013). The negative effects of metals might be associated with reduced viability of hemocytes, as was observed in *L. dispar* larvae fed with cadmium-supplemented feed (50 and 100 µg Cd/g of dry food). The tested larvae originated from both contaminated and not contaminated environments, but, DNA damage was noted only in those larvae from the environment not contaminated with cadmium, which proves that metal tolerance in insect population increases with the period of exposure to cadmium (Matića et al. 2016).

Some host plants of *S. exigua* are recognized as metal hyperaccumulators, of which certain species can accumulate high amounts of cadmium. One good example of metal hyperaccumulators may be *Zea mays* (maize) (Wang et al. 2007). Among the representatives of Brassicacae family, studies have widely investigated *Brassica juncea*, *B. oleracea*, *B. rapa*, *Arabidopsis halleri*, and *A. thaliana* (Nanda Kumar et al. 1995; Jahangir et al. 2008; Bhadkariya et al. 2014; Kazemi-Dinan et al. 2014).

When cadmium was present in larval food, the pupal weight was lower due to enhanced energy utilization during the larval period. This was due to the fact that there was cut energy input from the environment to larvae before change into the next developmental stage (Kooijman 2008). Luo et al. (2020) reported the consequences of cadmium exposure of larvae for further stages as well as for fecundity. Analyses on the imagoes of *S. exigua* (reared for over 130 generations) revealed differences in the reproduction process between cadmium strains and control strains. Cadmium accumulated in the tissues can interfere with physiological processes (e.g. vitellogenesis), causing a reduction in the amount of egg yolk and eggs produced. And, female *S. exigua* from the control strain laid twice as many eggs as those from the cadmium strain. Moreover, hatching of the laid eggs was less successful in cadmium strain (Płachetka-Bożek et al. 2018).

Sporadic incidents of starvation of herbivorous pests occur quite often (due to food scarcity, enemy attack, etc.) (Chen and Ruberson 2008). The effect of 24-h starvation in the 3rd instar of *Trichoplusia ni* and cadmium on insect development has been examined. The negative developmental effects of metal was only observed for those insects fed with artificial diet but not for those fed with natural host plants, cadmium hyperaccumulators (Konopka et al. 2013). Starvation and poisoning may lead to significant accumulation of energy sources in insects. The relative growth rate of *L. dispar* was dependent on the concentration of cadmium in the diet, with a decreased growth rate observed in insects exposed to a higher concentration of cadmium (Vlahović et al. 2001).

Thus, this study aimed to assess whether an insect population with a history of long metal exposure may better manage starvation and metal stress, referring to use of energy substrate sand effects on different developmental stages.

## Materials and methods

Two strains (control and cadmium) of *S. exigua* (beet armyworm) individuals (3rd instar) were obtained from the Institute of Biology, Biotechnology and Environmental Protection, University of Silesia, Katowice, Poland (temperature: 25 ± 1 °C, photoperiod: 16:8 L:D). The strains were reared for over 200 generations. The cadmium strain was selected using a semisynthetic diet supplemented with Cd at subtoxic doses—below the threshold of toxicity (44 mg/kg dry weight of the diet). The administered Cd may be available in the contaminated or metalliferous environments and is a result of earlier examinations to obtain mild mortality of larvae but significantly higher than among larvae kept in control conditions (Kafel et al., 2012; Wang et al. 2009). Larvae originating from control strain were divided into two groups: one group was fed with control diet (C group) and other group with a diet supplemented with cadmium (CCd group). A third group included larvae originating from the cadmium strain and fed with cadmium-contaminated diet (Cd group). The tested larvae were fed with a semisynthetic diet (prepared using wheat germ as the base and Brewer’s yeast) ad libitum that was not contaminated or contaminated with cadmium: 44 mg/kg dry weight of the diet. Ten larvae were grown in each Petri dish (with a diameter of 90 mm). In addition, the experimental groups were divided further and some 1-day old 3rd instars were subjected to 1-day starvation. During the examination of the concentration of biomolecules, 6 replications were made for each experimental group and for the total antioxidant capacity and the lipid peroxidation measurements—5 repetitions. The model of the experiment, referring to starvation and Cd treatment, is described in Table 1.

**Table 1:** Experimental groups and their history of cadmium treatment and period of starvation

Name of groups	C NS	C S	CCd NS	CCd S	Cd NS	Cd S
Strain origin	Control – optimal diet for over 200 generations		Control – optimal diet for over 200 generations		Treated with sublethal concentration of Cd for over 200 generations	
Feeding of larvae in the examined generation	With optimal diet		With diet containing sublethal concentration of Cd		With diet containing sublethal concentration of Cd	
Starvation period at the beginning of the 3rd stage	No	Yes	No	Yes	No	Yes

Body weight was determined for six individuals from each experimental group, at the beginning of the 5th instar (L5) and pupal stage.

### Sample preparation

Six individuals (same age as was mentioned for body weight measurement) were selected from each group to examine the concentration of energy substrates, the total antioxidant capacity (TAC), and level of malonyl aldehyde. To determine the concentration of protein, carbohydrates, total lipids, and glycogen, individuals were homogenized in aqueous lysis buffer containing 100 mM KH_2_PO_4_, 1 mM dithiothreitol, and 1 mM ethylenediaminetetraacetic acid with pH 5.0 (15 mg tissue in 180 mL buffer). For the determination of TAC and lipid peroxidation (malondialdehyde, MDA), individuals were homogenized in phosphate-buffered saline (PBS) with the concentration of 0.05 M and pH 7.4 (Foray et al. 2012).

### Estimation of biomolecule concentrations

After sample homogenization, 5 µL of supernatant was taken and protein content was determined by the Bradford (1976) method using bovine albumin as standard.

The content of carbohydrates, lipids, and glycogen was determined using a modified method described by Foray et al. (2012). Briefly, 180 µL of the homogenate was mixed with 20 µL of 20% Na_2_SO_4_ and 1500 µL of chloroform–methanol solution (1:2, v/v). Then, the mixture was centrifuged at 180 *g* for 15 min at 4 °C. The pellet was used later for glycogen determination, while the supernatant was used to determine the content of total carbohydrates and lipids. For estimating the content of carbohydrates, 150 µL of supernatant was evaporated at room temperature to a volume of about 30 µL, and then 240 µL of anthrone reagent was added. After heating the sample for 15 min at 90 °C, its absorbance was measured at 625 nm with D-glucose as a standard.

The total lipid content was measured using the classical method with vanillin reagent. After 100 µL of the sample had completely evaporated at 90 °C, 10 µL of 98% H_2_SO_4_ was added. The samples were incubated at 90 °C for 2 min and then cooled on ice. Subsequently, 190 µL of fresh vanillin dissolved in 68% orthophosphoric acid was added, and the samples were incubated at room temperature for 15 min. After incubation, the absorbance of the samples was measured at 525 nm using triolein as a standard (Foray et al. 2012).

To determine the content of glycogen, the pellet was first washed with 400 µL of 80% methanol, and then centrifuged at 16,000 *g* for 5 min at 4 °C. This step was repeated twice. Next, 1 mL of anthrone reagent was added and the pellet was incubated at 90 °C for 15 min. After cooling the samples on ice, absorbance was measured at 625 nm. A standard curve was plotted based on a standard solution of glucose (Foray et al. 2012).

### Total antioxidant capacity and lipid peroxidation measurements

Five larvae of 4th stage (L4) were selected from each experimental group. Each larva was homogenized in 0.05 M PBS (phosphate-buffered saline), pH 7.4, and the homogenate was centrifuged at 16,000 *g* for 5 min at 4 °C.

TAC was measured using the method described by Kafel et al. (2012). The antioxidant content was expressed in µmol Trolox/mg protein.

To measure the concentration of MDA, 50 µL of sample was mixed with 50 µL of 10% of trichloroacetic acid (TCA). Then, the mixture was centrifuged at 12,000 *g* for 10 min at 4 °C. After centrifugation, 70 µL of 1% of 2-thiobarbituric acid (TBA) was added to 90 µL of the supernatant and the mixture was vortexed. Then, the samples were heated at 100 °C for 60 min. After cooling the samples, their absorbance was read at 535 nm. The obtained results were expressed in µM MDA/mg protein (Bar-Or et al. 2001).

### Statistical calculations

Analysis of the obtained data and interpretation of the results were performed in Statistica 13.1. As testing of homogeneity of variance produced negative results, data were analyzed using the nonparametric Kruskal–Wallis test (differences were considered statistically significant at *p* < 0.05). Pearson correlation was calculated between biomolecule concentrations.

## Results

Among 1-day-old larvae of the 5th stage as well as pupae, the lowest body weight was noted for those from cadmium strain, which was starved at the 3rd larval stage. In the pupa case, also unstarved differed in body weight, an opposite tendency was observed—the lowest body weight was observed in the pupae from control strain. When starved and unstarved individuals were compared, differences were found among larvae from cadmium strain: individuals from the starved group were lighter (Table 2).

**Table 2:** Body weight [mg ±SD] of *S. exigua* individuals at different ages: 1-day old, 5th larval stage, and pupae. Different letters indicate significant differences between different groups with respect to the history of cadmium exposure; *significant differences between different groups with respect to the starvation period; Kruskal–Wallis, *p* < 0.05, *n* = 6

Starvation for 1 day		No		Yes	
Developmental stages		L5	P	L5	P
Strain origin and Cd treatment in the examined generation	C	70.6 ± 47.6A	51.6 ± 7.3A	113.9 ± 17.1A	60.4 ± 4.5A
	CCd	120.1 ± 48.1A	58.5 ± 7.6 AB	112.5 ± 11.0A	69.0 ± 7.0 AB
	Cd	155.6 ± 61.4A*	75.4 ± 10.2 B	56.3 ± 17.5 B*	70.0 ± 4.0 B

**Fig. 1:**
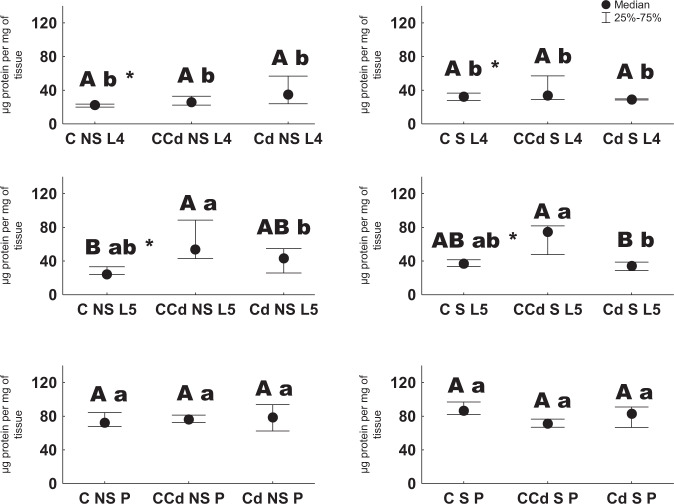
Protein concentration [µg/mg of tissue ±SD] in the 4th and 5th larval stages and pupae (different big letters indicate significant differences between different groups with respect to the history of cadmium exposure; different small letters indicate significant differences between different groups with respect to the age of animals; *significant differences between different groups with respect to the starvation period; Kruskal–Wallis, *p* < 0.05, *n* = 6)

When groups subjected to cadmium treatment were compared, larvae of the 4th stage had similar concentrations of proteins, regardless of whether they were unstarved or starved in the 3rd larval stage. However, when the 4th instars from the control strain were compared, those that were starved had a higher concentration of biomolecules than the unstarved. A similar relationship was observed for larvae of the 5th stage from control strain. Regardless of starvation, the 5th stage larvae from control strain and exposed to cadmium had high concentration of proteins compared to other groups. Pupae from different groups had similar protein concentrations. Individuals from pupal stage mostly had higher content of proteins than the 4th instars, while pupae from the starved cadmium treated control group had lower content of protein compared to the larvae (Fig. 1).

**Fig. 2:**
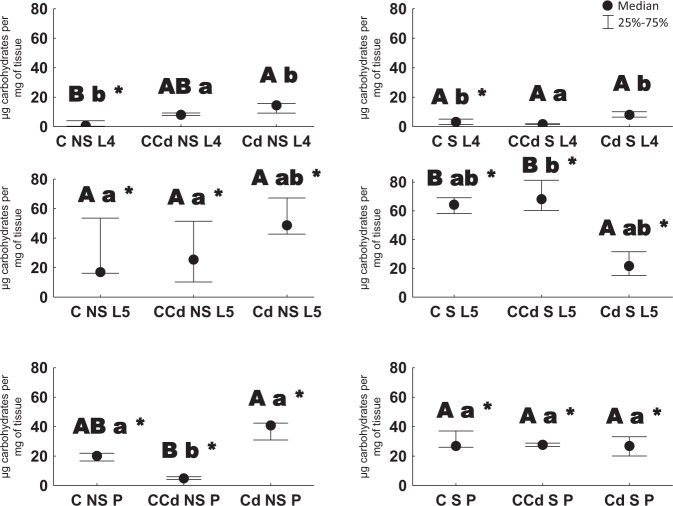
Carbohydrate concentration [µg/mg of tissue ±SD] in the 4th and 5th larval stages and pupae (description as in Fig. 1, Kruskal–Wallis test, *p* < 0.05)

Among the groups not subjected to starvation, an enhanced level of carbohydrates was noted in individuals of cadmium strain compared to those from control strain (in the case of L4 larvae: C NS L4 versus Cd NS L4, and pupae: CCd NS P versus Cd NS P). Among groups subjected to starvation, an opposite tendency was observed for 5th stage larvae. Larvae of 4th and 5th stage from control strain (not exposed and exposed to Cd) had a higher level of carbohydrates when starved (the only exception was the lack of difference between CCd NS L4 and CCd S L4). In the case of larvae of the 5th stage and pupae from cadmium strain, a contrasting relationship was found between starved and earlier unstarved individuals. Regarding changes with respect to age, the highest level of biomolecules was noted in pupae compared to 4th instars (Fig. 2).

**Fig. 3:**
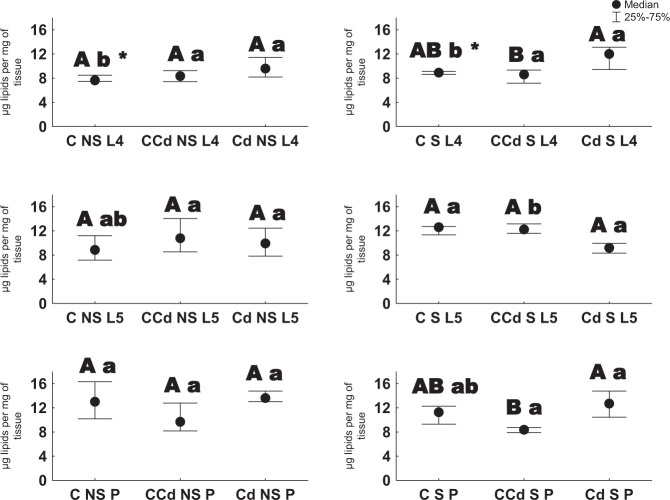
Lipid concentration [µg/mg of tissue ±SD] in the 4th and 5th larval stages and pupae (description as in Fig. 1, Kruskal–Wallis test, *p* < 0.05)

In general, a slight variation in lipid concentration was observed in individuals from the examined groups. Among the groups with earlier starved individuals and exposed to Cd, a higher lipid concentration was established in cadmium strain individuals compared to control individuals (the 5 h instars and pupae). Between starved and unstarved individuals, the only difference was found in the case of 4th instars of control group. With respect to age, the main differences were found in control strain individuals—a lower level of lipids was observed in the 4th instars (Fig. 3).

**Fig. 4:**
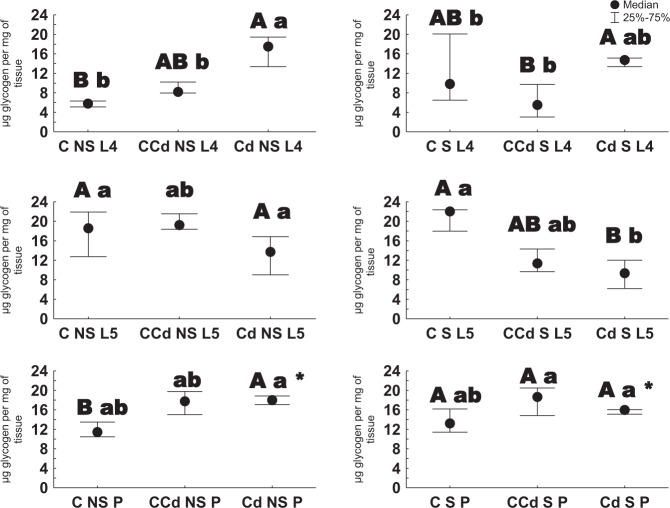
Glycogen concentration [µg/mg of tissue ±SD] in the 4th and 5th larval stages and pupae (description as in Fig. 1, Kruskal–Wallis test, *p* < 0.05)

When considering glycogen level, the varied effect of cadmium between different aged individuals was observed. Among those unstarved, cadmium strain individuals had higher amounts of glycogen than the control ones (larvae of the 4th stage and pupae). For the earlier starved animals, the 4th instars from control strain exposed to Cd had a lower concentration of glycogen compared to those from cadmium strain. And, the 5th stage larvae from cadmium strain exhibited low concentration of the biomolecule compared to those not exposed to metal. When compared individuals from starved and unstarved groups, the differences were found only for pupae from cadmium strain. In terms of age, a lower glycogen concentration was generally found in the 4th instars (groups: CNS, CCdNS; CS, CCdS, CdCdS). Among cadmium strain individuals that earlier experienced 1-day starvation, a lower glycogen concentration was observed in the 5th instars compared to pupae (Fig. 4).

**Fig. 5:**
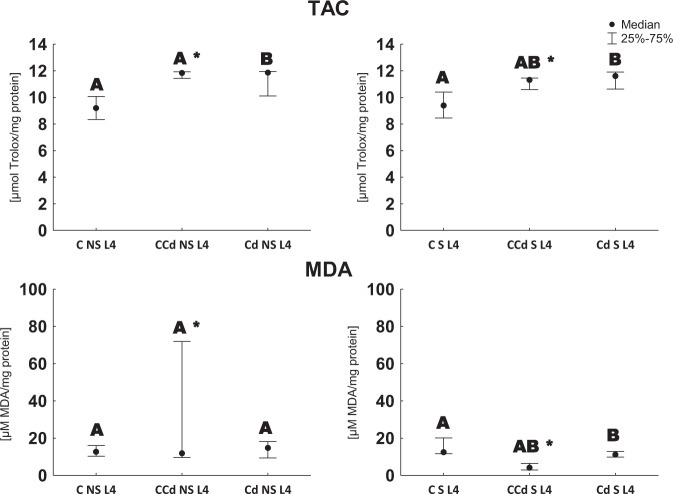
TAC [µmol/mg protein] and lipid peroxidation level [µM MDA/mg protein ±SD] in the 4th larval stage (description as in Fig. 1, Kruskal–Wallis test, *p* < 0.05, *n* = 5)

Among the 4th stage larvae, TAC was higher in cadmium strain compared to the control ones. In the previously starved cadmium-exposed controls, TAC was found to be higher compared to those unstarved. A higher amount of MDA, produced in the ongoing lipid peroxidation process, was found in the 4th instars from the control strain exposed to cadmium, compared to the control group (but only in the individuals previously subjected to starvation). Among the larvae from the control group exposed to cadmium, a higher content of MDA was noted in unstarved larvae compared to the starved ones (Fig. 5).

## Discussion

In this study, starvation differently affected control and cadmium strain individuals. In the case of 5th instars, differences were noted only in individuals not subjected to starvation, with heavier individuals found from the control strain (Table 2). Studies on *L. dispar*, another representative of Lepidoptera, have also revealed a decrease in the larval body mass under Cd exposure (Vlachović et al. 2017). Earlier studies on *S. exigua* have demonstrated the effects on cadmium exposure, but no effects on pupal body weight, independent of sex (Kafel et al. 2021). In this study, regardless of sex, cadmium strain pupae were heavier compared to control strain ones, independent of 1-day starvation. It suggested that cadmium strain individual may effectively compensate the effect of metal exposure. When, as reported by Chen and Ruberson (2008), the time of development was prolonged, pupal weight was changed, especially when there was starvation for 2 days at earlier larval stages and also at the 3rd stage. In our study, starvation lasted for only 1 day and pupal weight was found to be unaffected regardless of strain origin. By contrast, Fisher, Fieldler (2001) showed that 2-day starvation of the last larval instars of *Lycaena tityrus* significantly affected their further development: the pupae body weight decreased and maturation was delayed. These changes may have a negative impact on the reproduction strategies (Płachetka-Bożek et al. 2018). Exposure to cadmium changed the weight of larvae “but not adults” in the case of the dipteran representative *Chrysomya megacephala*. This effect was also accompanied by prolonged development and enhanced mortality (Singh and Kaur 2017). These findings suggest that the effects of starvation might depend not only on species but also on the period of starvation and the developmental stage at which starvation occurred.

Bednarska et al. (2012) showed that ground beetles living in an area with high availability of metals, such as Cd and Zn, were characterized by increased energy expenses, which affected the content of energy substrates. Kafel et al. (2021) revealed that, depending on age, individuals from control and cadmium strain showed differences in the content of energetic substrates when exposed to metal. This finding was partly confirmed in the present study—the concentration of protein, carbohydrates, and glycogen was found to be lower in 4th instars compared to individuals of other ages (5th instars and pupae).

Among the individuals originating from the control strain and exposed to Cd (both starved and unstarved), a higher protein concentration was observed only in 5th instars. This may be related to elevated defense with protein partition. Such an assumption was also put forth by Kafel et al. (2021) who reported that higher protein concentration could be attributed to higher engagement or turnover of proteins during defense processes.

As presented by Kafel et al. (2021), the content of lipids may be influenced by Cd exposure and possibly higher utilization of lipids causes a reduction in their availability in larval bodies, especially in the case of cadmium strain individuals. It is interesting that 1-day starvation did not seem to affect the concentration of total lipids in the larvae of *S. exigua* in the present study. But, the usage of soluble carbohydrates had been changed, especially after starvation incident. Such effect differed individuals from cadmium and control strains. In cadmium strain individuals, starvation may be associated with lower carbohydrate concentration (L5 and P), so the fast energy expenses connected with soluble sugars usage seemed to be higher than in control strain individuals. But, such situation did not affect body mass rate and the cadmium pupae had high body mass of the pupae. A previous study also showed that cadmium influenced the whole-body lipid content of the larvae and pupae of beekeeping pest *Galleria mellonela* (Shin et al. 2001). Of course, the content of different lipids may be differently affected; for example, in the 3rd instar of *Helicoverpa armigera*, the content of cholesterol was enhanced while that of triglycerides was decreased under the influence of cadmium (Baghban et al. 2014). The effect of cadmium toxicity on carbohydrate metabolism has already been established (Ilijin et al. 2010; Kafel et al. 2021), and this dependence was also observed in the present study for 1-day starvation. Starvation caused a decrease in the availability of sugars in the cadmium strain individuals, while the effect was opposite in unstarved individuals. A decrease in carbohydrate concentration was noted with prolonged starvation (from 8 to 72 h) in the study on *H. axyridis* individuals (Shi et al. 2017). The authors noted that a shorter starvation period was associated with higher utilization of glycogen, with differences in glycogen level observed between starved and unstarved pupae only in the case of cadmium strain. It was suggested that energy availability can limit the ability of organisms to survive under stress. Stress may play an important role in the physiological adaptation of insects. This was confirmed by a study on flies (members of the *Drosophila genus*) that were not only starved but also desiccated. The authors reported that the most sensitive responses were manifested by changes in carbohydrate concentration (Marron et al. 2003). When insects are subjected to starvation and exposed to cadmium, the carbohydrate concentration in their bodies changes, which is considered to be the most sensitive biomarker.

The present study showed an increase in TAC in individuals from cadmium strain (both starved and unstarved). Farahani et al. (2020) showed that different stressors such as starvation (24 and 48 h), temperature, or parasitism led to an increase in antioxidant defense in the last larval instar of carob moth *Ectomyelois ceratiniae*. Cadmium exposure may lead to enhanced energy expenditure related to the elevation of antioxidant response and also increased lipid peroxidation (e.g. *S. litura* larvae; Suganya et al. 2016). However, in this study, lipid peroxidation was found to be higher in control than starved cadmium strain individuals.

Strategies to diminish the negative action of cadmium were more effective in *S. exigua* larvae from the cadmium strain (Kafel et al. 2012, 2014). In the present study, it was difficult to interpret this effect based on a comparison of individuals from two (cadmium and control) strains; however, their responses were visibly different. Moreover, responses with respect to the availability of energetic substances might change when individuals in earlier 3rd stage are subjected to short-term starvation.

In conclusion, control and cadmium strain animals differed in gained pupal body mass. Cadmium strain individuals seem to better cope with stressors despite oxidative stress evidence—lipid peroxidation enhancement and higher soluble carbohydrate losses. It is worthwhile to highlight the changes in carbohydrate concentration as possible sensitive biomarker of metal and starvation stress, but varied between different strain individuals. Energy expense possibly also modified the usage of the other analyzed biomolecules: proteins glycogen or lipids, but in a strain dependent mode.
